# John Sims

**Published:** 1838-10

**Authors:** 


### OBITUARY.

### JOHN SIMS, M.D.

Died, on Thursday, the 19th July, at seven P.M., aged forty-six, Dr. Sims, of 37, Cavendish square, one of the most zealous and disinterested members of the medical profession, to which he may be said to have fallen a sacrifice. About six years ago he had a most dangerous illness, produced by the absorption of poison while dissecting, during the prosecution of researches on morbid anatomy; a study in which he was much interested. From this severe attack he narrowly escaped. The result of some of his subsequent labours was a series of valuable papers on the morbid anatomy of the brain, which were read at the Medical and Chirurgical Society, and published in a late volume of the Transactions of that Society. The attack which proved fatal was a malignant fever of a low typhoid character, which he is supposed to have caught at the St. Marylebone Infirmary, to which institution he has for many years been physician. The skill and disinterested kindness which he displayed in the execution of his duties at the infirmary, as well as to gratuitous patients in his private practice, procured for him universal respect and esteem.

